# Antimicrobial resistance survey and whole-genome analysis of nosocomial *P. Aeruginosa* isolated from eastern Province of China in 2016–2021

**DOI:** 10.1186/s12941-023-00656-1

**Published:** 2024-02-09

**Authors:** Zimeng Hu, Lu Zhou, Xingyu Tao, Pei Li, Xiangkuan Zheng, Wei Zhang, Zhongming Tan

**Affiliations:** 1 College of Veterinary Medicine, Nanjing Agricultural University, No.1 Weigang, Xuanwu District, Nanjing City, Jiangsu Province 210095 People’s Republic of China; 2 Key Lab of Animal Bacteriology, Ministry of Agriculture, Nanjing, 210095 China; 3 Sanya Institute of Nanjing Agricultural University, Sanya, 572024 China; 4 Department of Acute Infectious Disease Prevention and Control, Jiangsu Provincial Center for Disease Prevention and Control, Nanjing, 210009 China; 5 NHC Key Laboratory of Enteric Pathogenic Microbiology, Jiangsu Provincial Center for Disease Control and Prevention, Nanjing, 210009 China

**Keywords:** *P. Aeruginosa*, Multiple drug resistance, Pan-genomic analysis, ExoU, CrpP

## Abstract

**Background:**

*Pseudomonas aeruginosa* is a major Gram-negative pathogen that can exacerbate lung infections in the patients with cystic fibrosis, which can ultimately lead to death.

**Methods:**

From 2016 to 2021, 103 strains of *P. aeruginosa* were isolated from hospitals and 20 antibiotics were used for antimicrobial susceptibility determination. Using next-generation genome sequencing technology, these strains were sequenced and analyzed in terms of serotypes, ST types, and resistance genes for epidemiological investigation.

**Results:**

The age distribution of patients ranged from 10 days to 94 years with a median age of 69 years old. The strains were mainly isolated from sputum (72 strains, 69.9%) and blood (14 strains, 13.6%). The size of these genomes ranged from 6.2 Mb to 7.4 Mb, with a mean value of 6.5 Mb. In addition to eight antibiotics that show inherent resistance to *P. aeruginosa*, the sensitivity rates for colistin, amikacin, gentamicin, ceftazidime, piperacillin, piperacillin-tazobactam, ciprofloxacin, meropenem, aztreonam, imipenem, cefepime and levofloxacin were 100%, 95.15%, 86.41%, 72.82%, 71.84%, 69.90%, 55.34%, 52.43%, 50.49%, 50.49%, 49.51% and 47.57% respectively, and the carriage rate of MDR strains was 30.69% (31/101). Whole-genome analysis showed that a total of 50 ST types were identified, with ST244 (5/103) and ST1076 (4/103) having a more pronounced distribution advantage. Serotype predictions showed that O6 accounted for 29.13% (30/103), O11 for 23.30% (24/103), O2 for 18.45% (19/103), and O1 for 11.65% (12/103) of the highest proportions. Notably, we found a significantly higher proportion of ExoU in *P. aeruginosa* strains of serotype O11 than in other cytotoxic exoenzyme positive strains. In addition to this, a total of 47 *crpP* genes that mediate resistance to fluoroquinolones antibiotics were found distributed on 43 *P. aeruginosa* strains, and 10 new variants of CrpP were identified, named 1.33, 1.34, 1.35, 1.36, 1.37, 1.38, 1.39, 1.40, 1.41 and 7.1.

**Conclusions:**

We investigated the antibiotic susceptibility of clinical isolates of *P. aeruginosa* and genomically enriched the diversity of *P. aeruginosa* for its prophylactic and therapeutic value.

**Supplementary Information:**

The online version contains supplementary material available at 10.1186/s12941-023-00656-1.

## Introduction

*Pseudomonas aeruginosa* is a Gram-negative, opportunistic human pathogen that is considered one of the major pathogens associated with hospital-acquired infections, infecting cystic fibrosis lungs and promoting an accelerated decline in lung function [1]. The threat of morbidity and mortality from hospital-acquired infections caused by multidrug-resistant (MDR) or extensively-drug resistant (XDR) strains of *P. aeruginosa* has increased significantly [2]. Meanwhile, the complex genome of *P. aeruginosa* also confers resistance to antibiotics, usually exhibits a high degree of intrinsic resistance to β-lactams, fluoroquinolones, and aminoglycoside antibiotics [3].

The use of genomics to analyze ST types, serotypes, resistance genes and virulence genes of *P. aeruginosa* is a very effective method for the prevention and control of *P. aeruginosa* epidemics. Currently, a number of high-risk clonal strains (e.g. ST111, ST175, ST235) with strong global transmissibility, usually MDR/XDR strains, and a defined ability to spread and cause serious infections [2, 4]. Several studies have shown that isolates belonging to serotypes O1, O6, O11 and O12 accounted for more than 65% of *P. aeruginosa* infections [5, 6]; serotypes O4 and O12 isolates are usually associated with resistance to various antibiotics [7, 8]; serotypes O5, O6 and O11 are commonly found in burn wound infections [9]; serotype O11 has also been reported to be strongly associated in MDR-positive strains with ExoU-positive strains [10, 11].

The genome size of *P. aeruginosa* typically ranges from 5.5 Mb to 7 Mb, and its large genome also confers the ability to survive in a wide range of environments [3]. The *P. aeruginosa* pan-genome consists of a “core genome” and a “dispensable genome”, and a core genome containing genes present in all strains, and “dispensable genome”: a composition of genes unique to each strain [12]. Dispensable genomes typically consist of horizontally transferable elements, which include integrative and conjugative elements (ICEs), genomic islands (GIs), prophages, transposons, insertion sequences (ISs), and integrons [3]. CrpP, an enzyme capable of phosphorylating ciprofloxacin, was described in 2018 as encoded in plasmid pUM505 from *P. aeruginosa*; cloning into J53 increased the MIC to ciprofloxacin from 0.008 mg/L to 0.06 mg/L. Since then, crpP-like genes have been reported in *E. coli*, *K. pneumoniae* and *P. aeruginosa* [13–19]. Although there is controversy as to whether CrpP can be investigated against fluoroquinolone antibiotics, it is still important to carry out investigations of its related variants.

In this research, we studied 103 strains of *P. aeruginosa* isolated from Jiangsu Province from 2016 to 2021 for resistance phenotypes, antimicrobial resistance genes, pan-genome analyse, ST types, serotypes, investigate the association between cytotoxic exoenzyme and serotypes, and emphasize the diversity of CrpP protein and integration and conjugation elements (ICE) carrying CrpP protein, which will benefit our understanding of *P. aeruginosa* transmission.

## Materials and methods

### Bacterial strains, media, and culture conditions

In 2016–2021, 103 strains of *P. aeruginosa* were isolated from patients in various hospitals in Jiangsu Province, China. *P. aeruginosa* was grown in Luria-Bertani (LB) medium at 37 °C. ATCC27853 was used as the control strain for the drug sensitivity test.

### Antimicrobial susceptibility of *P. Aeruginosa* isolates

20 antibiotics were selected including amikacin, amoxicillin-clavulanate, ampicillin, ampicillin-sulbactam, aztreonam, cefazolin, cefepime, cefotaxime, ceftazidime, chloramphenicol, ciprofloxacin, gentamicin, imipenem, levofloxacin, meropenem, moxifloxacin, piperacillin, piperacillin-tazobactam, tetracycline, trimethoprim-sulfamethoxazole were tested by the agar dilution method. Results were determined according to the Clinical and Laboratory Standards Institute (CLSI, 2019) guidelines, in addition to eight of *P. aeruginosa* to antibiotics for which they carry an inherent resistance mechanism.

### Whole genome sequencing and analysis

103 strains of *P. aeruginosa* were sequenced in draft genome by Illumina NovaSeq PE150. CLC Genomics WorkBench software version 22.0 was used for the sequence assembly. Some strains carrying crpP-like genes are selected to use Oxford Nanopore Technology for third -generation sequencing. The bacterial genome was annotated by Prokka (v1.14.6)(https://github.com/tseemann/prokka) [20]. ABRicate (https://github.com/tseemann/abricate) was used for the prediction of antimicrobial resistance genes and virluence genes. Transposon analysis was predicted on the ICEfinder (https://bioinfo-mml.sjtu.edu.cn/ICEfinder/index.php) website, oriTfinder (https://tool-mml.sjtu.edu.cn/oriTfinder/oriTfinder.html) [21] were used to predict oriT and relaxase on transposon, and transposon gene clusters were mapped at GBKviz (https://moshi4-gbkviz-srcgbkvizgbkviz-webapp-vaurf6.streamlit.app/) and Inkscape. The evolutionary tree of CrpP amino acids was performed using MEGA X, with bootstrap set to 1000. Amino acid alignment of CrpP amino acids sequences was performed using Jalview [22]. MLST (multilocus sequence typing)(https://github.com/tseemann/mlst) was used to predict the ST type of *P. aeruginosa* isolates [23]. Phyloviz was used to perform the minimum spanning tree [24]. Pasty (https://github.com/rpetit3/pasty) was used for serotype prediction of *P. aeruginosa* [25, 26]. Pan-genomic analysis of the gff files generated from Prokka annotations was performed using Roary (https://github.com/ggh2020/Roary) [27]. The newick generated by the Roary annotation was used to generate the evolutionary tree at the chiplot (https://www.chiplot.online/) website. The size of the genome was statistically processed using seqkit (https://github.com/shenwei356/seqkit) [28]. Genetic distances between genomes were calculated by mash (https://github.com/marbl/Mash) [29].

## Results

### Bacterial source

Of the 103 *P. aeruginosa* strains isolated, the distribution of patient age ranged from 10 days to 94 years, with a median age of 69 years old. The isolates were mainly isolated from sputum (*n* = 72, 69.9%), blood (*n* = 14, 13.6%). Table 1 shows the distribution of the sources of the isolates and Attachment 1 provides all information on the isolates.

**Table 1:** Distribution of sources of isolated strains

Samples	Number	Site of separation
Sputum	72	
Blood	14	
Sanies	5	Isolated from patients presenting with fractures, left-sided nasal polyps, soft tissue infections, broken ear sores, and symptoms of respiratory distress without identifying the specific site of pus secretion
Secreta	3	Isolated from patients presenting with symptoms of finger injury, embolism and thrombosis, and compound trauma without identifying the specific site of pus secretion
Urine	3	urine
Bile	1	
Drainage liquid	1	
Faeces	1	Separated from patients presenting with diarrhea
Pleural effusion	1	

### Results of bacterial antibiotic sensitivity tests

**Fig. 1:**
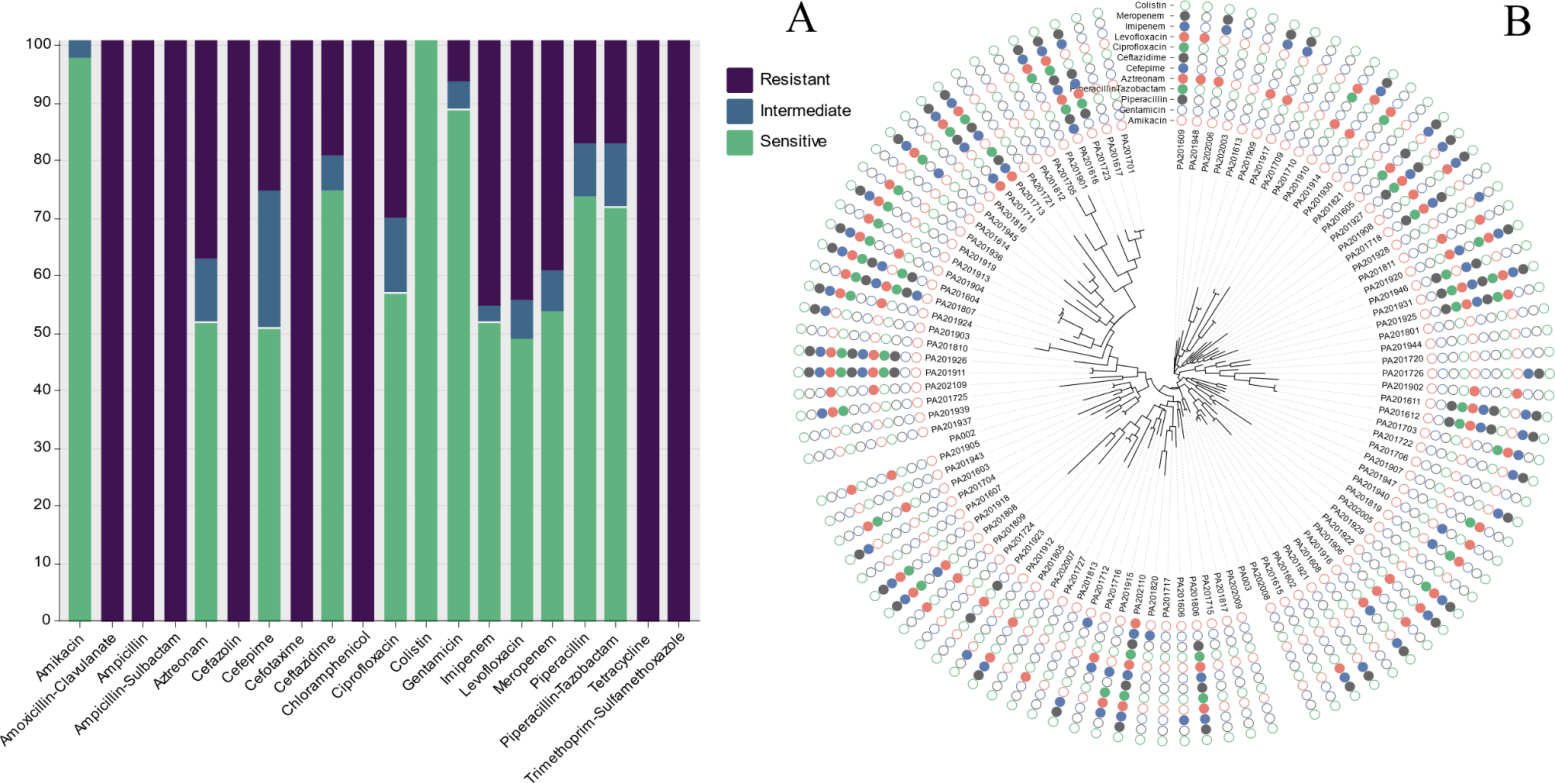
**A**: Results of the antimicrobial sensitivity test for *P. aeruginosa* strains, the x-axis is the type of antibiotic and the y-axis is the number of resistant strains of bacteria; **B**: Evolutionary tree of *P. aeruginosa* strains with solid filled colors as a result of drug resistant phenotypes, a total of 8 major antibiotic classes, including 12 antibiotics, were involved in the analysis, the evolutionary tree in the center of the circle graph was generated from the roary generated accessory_binary_genes.fa.newick file

*P. aeruginosa* strains were most resistant to amoxicillin-clavulanate, ampicillin, ampicillin-sulbactam, cefazolin, cefotaxime, chloramphenicol, tetracycline, trimethoprim-sulfamethoxazole, all with a resistance rate of 100%. The sensitivity rates for colistin, amikacin, gentamicin, ceftazidime, piperacillin, piperacillin-tazobactam, ciprofloxacin, meropenem, aztreonam, imipenem, cefepime and levofloxacin were 100%, 95.15%, 86.41%, 72.82%, 71.84%, 69.90%, 55.34%, 52.43%, 50.49%, 50.49%, 49.51% and 47.57% respectively (Fig. 1). The data from the antimicrobial sensitivity tests are listed in Attachment 2.

### Antimicrobial resistance genes, ST types, and serotypes

**Fig. 2:**
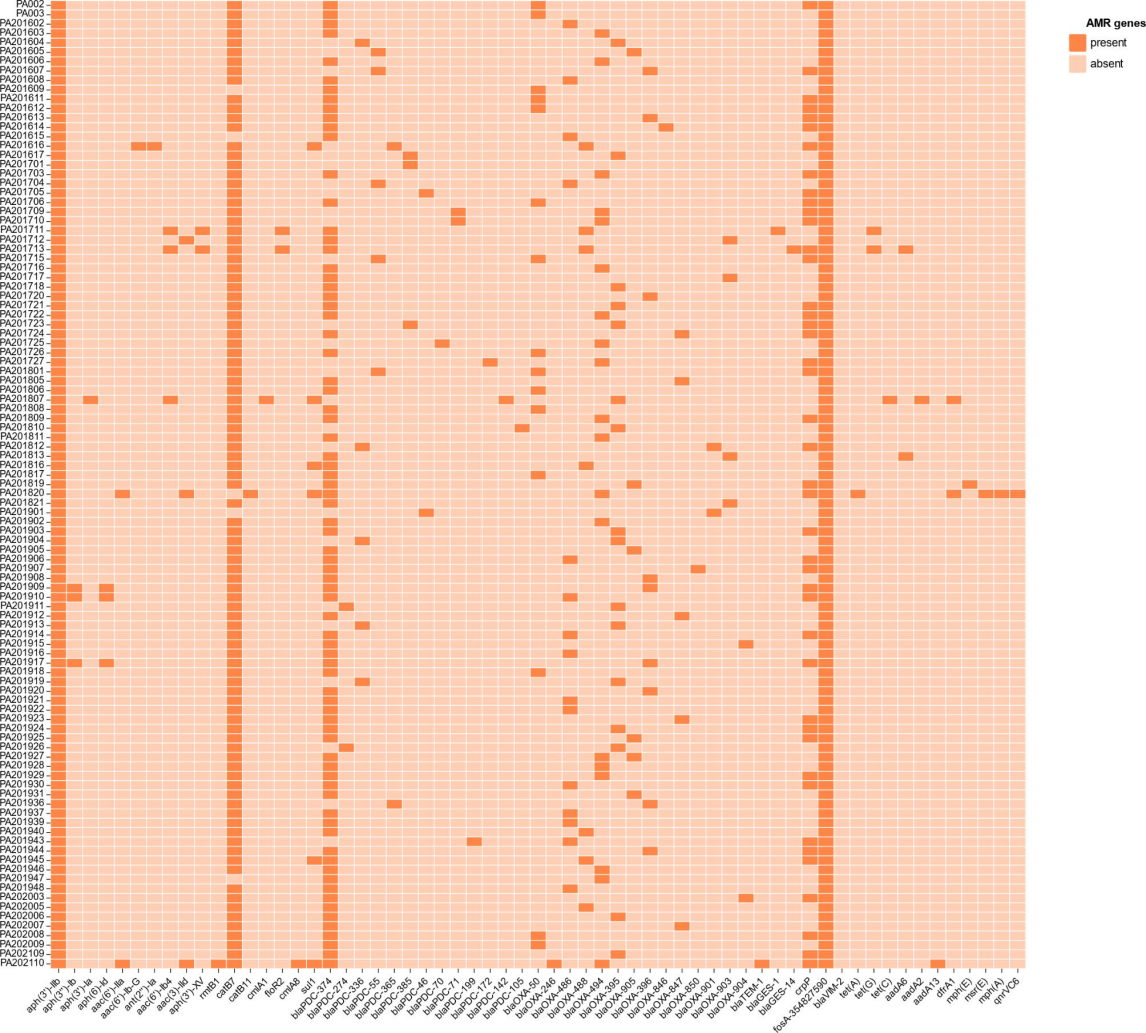
Presence and absence of antimicrobial resistance genes. The horizontal axis is the name of the resistance gene and the vertical axis is the name of the strain; present is labeled as dark orange and absent is labeled as light orange

A total of 61 resistance genes were identified in 103 *P. aeruginosa* strains. The top five resistance genes were *aph(3’)-IIb* (103, 103/103), *fosA* (103, 103/103), *catB7* (102, 102/103), *bla*_PDC−374_ (77, 77/103), and *crpP* (47,47/103) (Fig. 2).

**Fig. 3:**
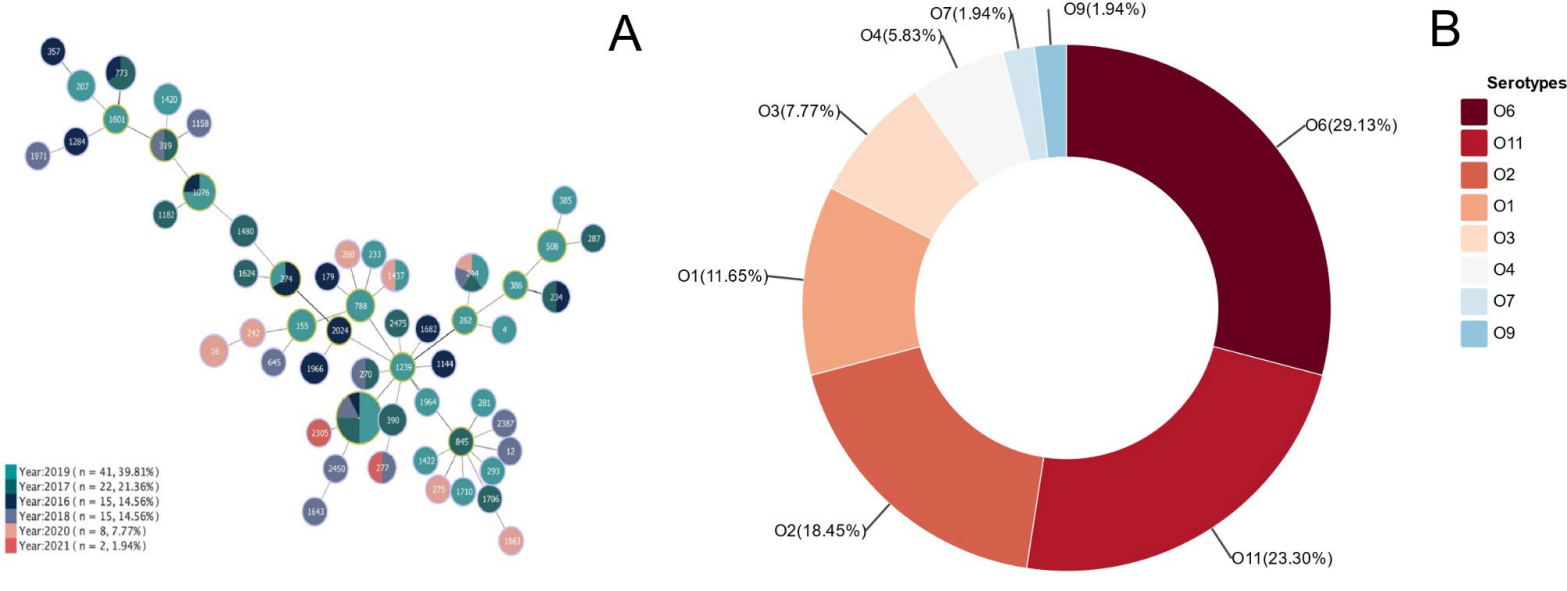
**A**: Distribution of *P. aeruginosa* isolates by ST type in six years. The years spanned from 2016 to 2021, with the highest number of occurrences in 2019, *n* = 41, representing 39.81%; **B**: The proportion of various serotypes. A total of 8 serotypes were predicted, of which 29.13% (30/103) were O6, 23.30% (24/103) were O11, 18.45% (19/103) were O2, 11.65% (12/103) were O1, 7.77% (8/103) were O3, 5.83% (6/103) were O4, 1.94% (2/103) were O7 and 1.94% (2/103) were O9

The results of ST type analysis showed that ST244 (5/103) was found in four separate years, except for the ST type which could not be identified. A total of 50 ST types were identified, and overall these 103 clinical *P. aeruginosa* isolates had a wide distribution of ST types. Minimum spanning tree plotting using phyloviz (Fig. 3).

### Carrying of extracellular enzymes in *P. Aeruginosa*

**Fig. 4:**
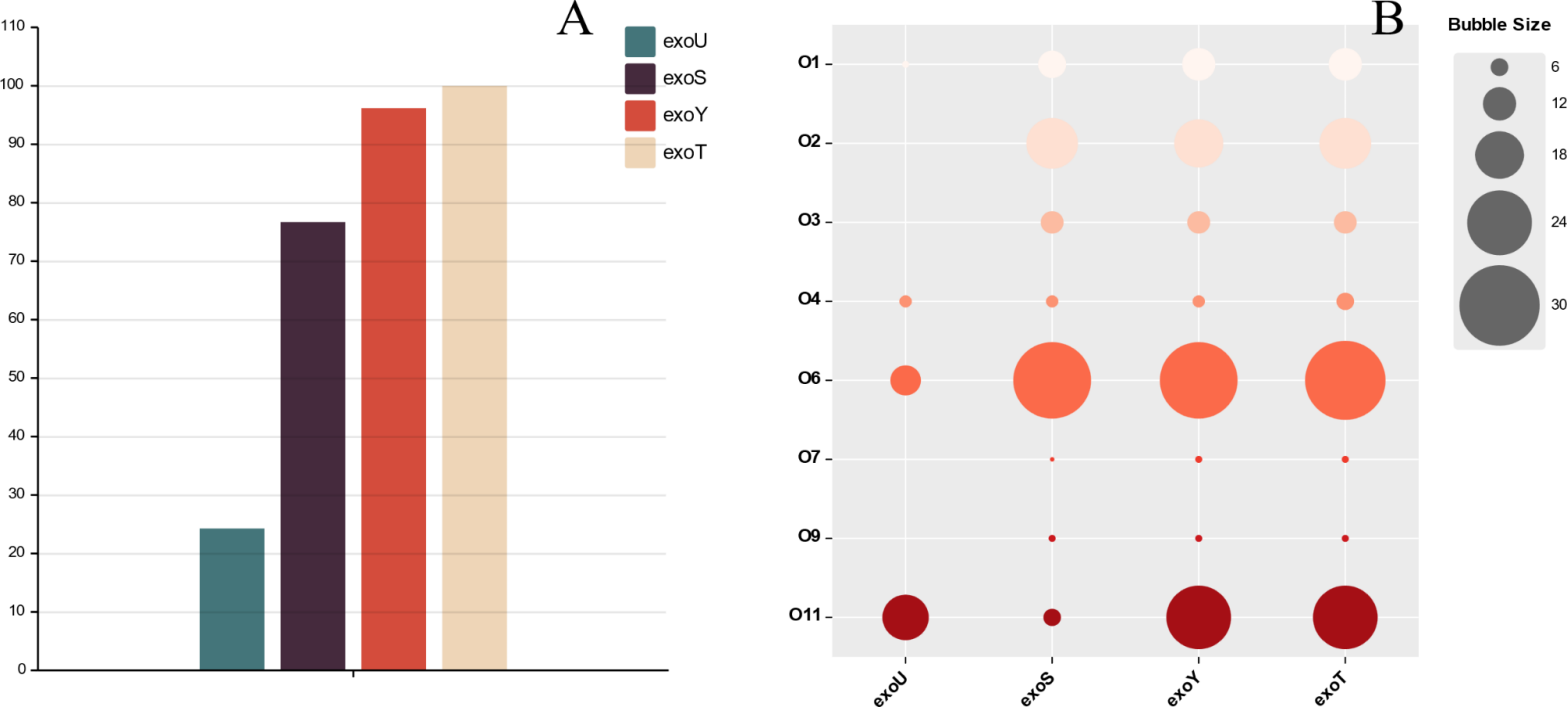
**A**: ExoU,ExoS,ExoY,ExoT among all *P. aeruginosa* strains, respectively, the y-axis is the number of strains; **B**: The proportion of O serotypes among different extracellular enzymes strains, the proportion of exoU-positive strains with serotype O11 was 50% (17/34), which is relatively large

The ExoT, ExoY, ExoS and ExoU proteins of the type III secretion system were the focus of attention in this article. The proportion of ExoU in 103 *P. aeruginosa* was 24.27% (25/103), ExoS 76.70% (79/103), ExoY 96.17% (99/103), and ExoT 100% (103/103) (Fig. 4A). Of the 34 ExoU-positive *P. aeruginosa* strains, the percentage of serotype O11 was 50% (17/34), O6 was 32.35% (11/34), O4 was 11.76 (4/34); meanwhile, among the ExoS-positive 79 *P. aeruginosa* strains, the percentage of serotype O6 was 36.71% (29/79), O6 was 24.05% (19/79) and O1 was 12.66% (10/79) (Fig. 4B).

### Pan-genome analysis

**Fig. 5:**
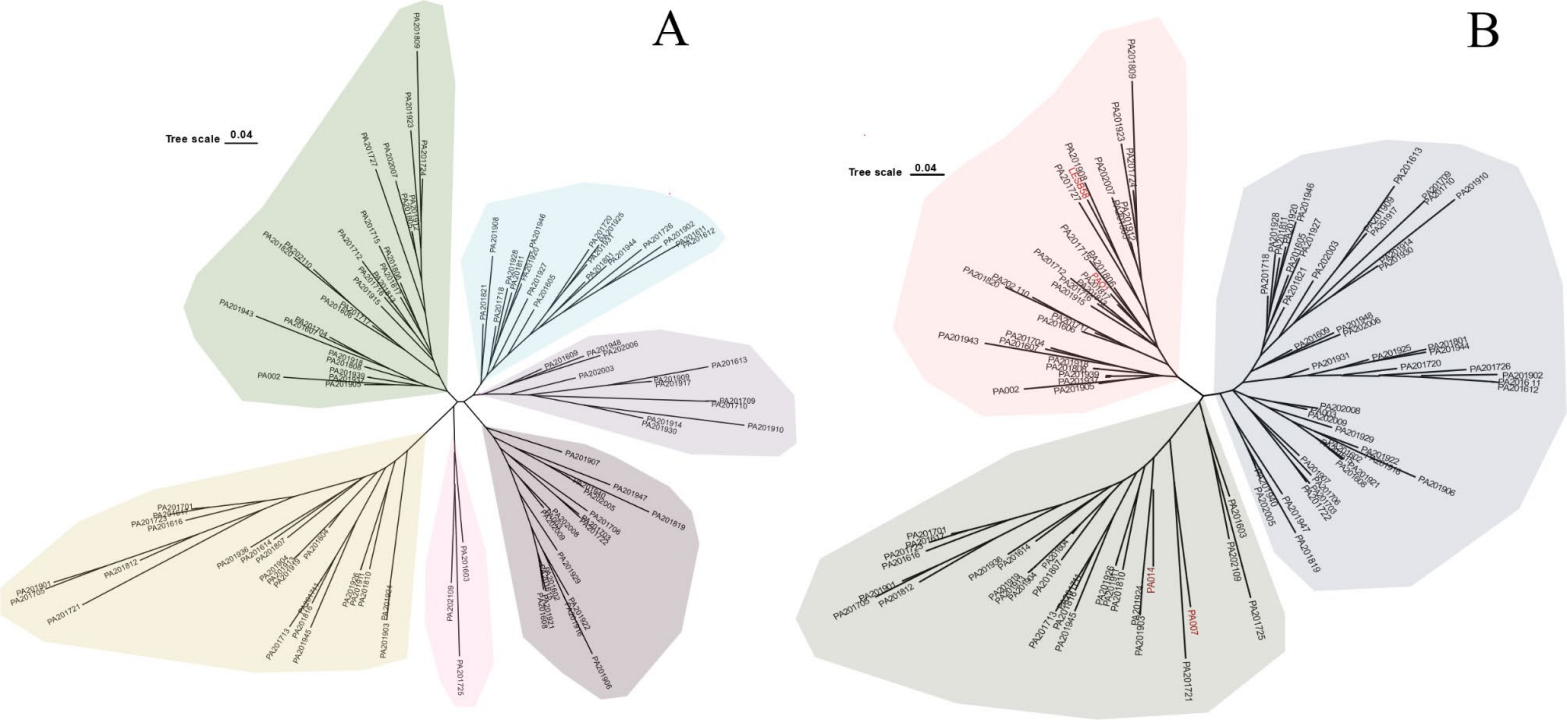
**A**: Clustering analysis of 103 *P. aeruginosa*, not added to the reference strains, was divided into a total of five large branches; **B**: Four representative strains of *P. aeruginosa* were introduced in 103 *P. aeruginosa* strains and 65 *P. aeruginosa* were clustered with the four representative strains within two branches

The main reference strain for genetic and functional studies of *P. aeruginosa*, PAO1 (ACCESSION: NC_002516), the “Liverpool endemic strain” *P. aeruginosa* LESB58 (NC_011770), which was found to be highly transmissible in cystic fibrosis patients, the ExoU-positive strain PA14 (NC_008463), the Argentine clinical isolate PA7 (NC_009656), which was reported to have an unusual antibiotic resistance pattern, were selected as reference strains with different characteristics for clustering analysis with the isolates (Fig. 5) [30].

**Fig. 6:**
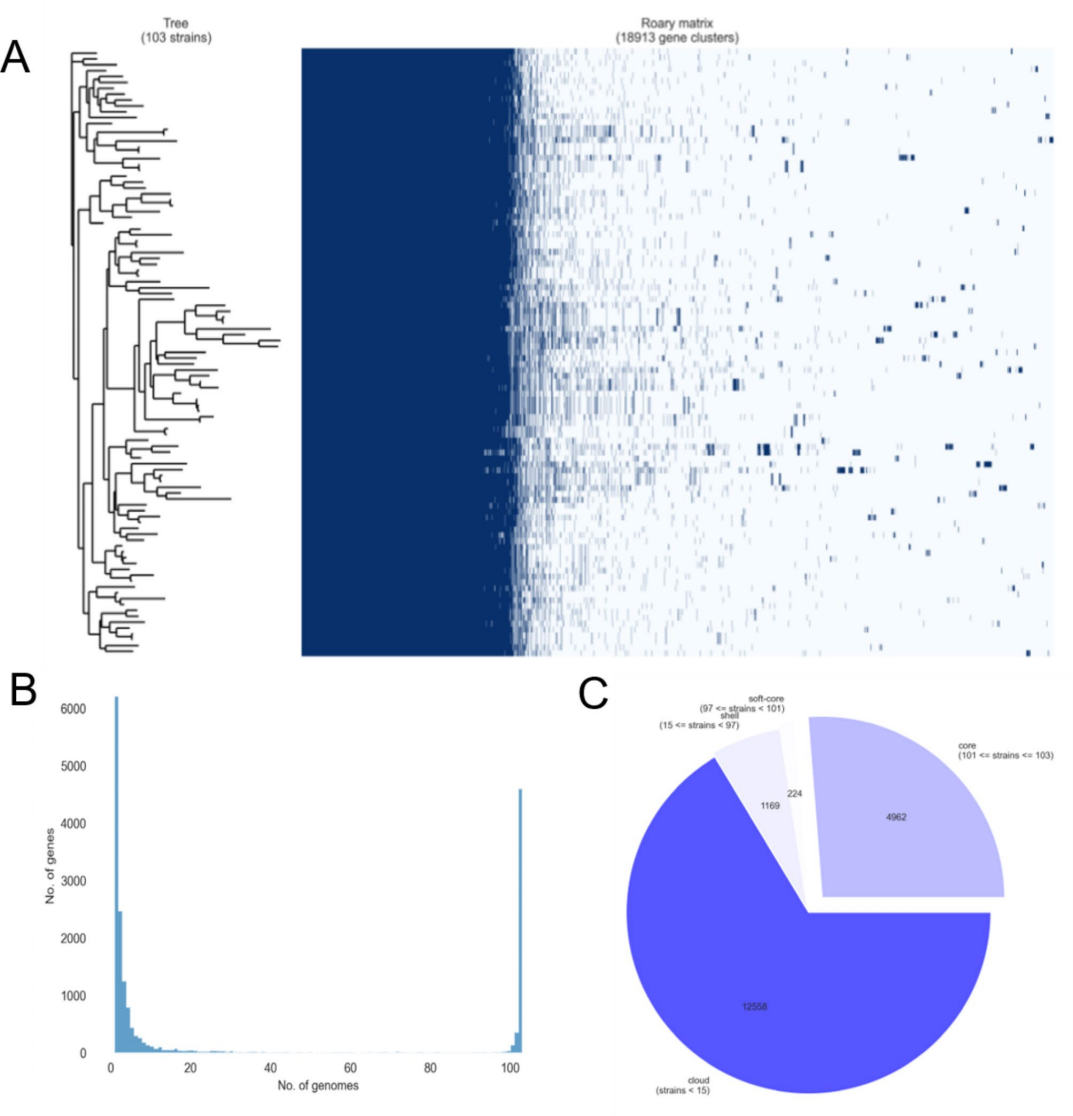
**A**: The presence and absence matrix of the 103 *P. aeruginosa* isolates, the dark blue color represents the presence of genes; **B**: Frequency of genes versus the number of genomes, the horizontal coordinate is the number of genomes involved in the analysis and the vertical coordinate is the total number of genes contained in a single bacterial strain; **C**: The number of core genes, soft-core genes, shell genes, cloud genes and its percentage

The sizes of these genomes ranged from 6,195,834 bp to 7,353,881 bp, with a mean value of 6,521,728 bp, and a total of seven sequences larger than 7 Mb. A core genome alignment was generated with Roary, the total number of genes in the 103 *P. aeruginosa* is 18,913. 4,962 core genes (26.24% of all isolates), soft core genes (1.18% of all isolates) comprising 224 genes, 1,169 shell genes (6.18% of all isolates) and 12,558 cloud genes (66.40% of all isolates) were identified (Fig. 6).

**Fig. 7:**
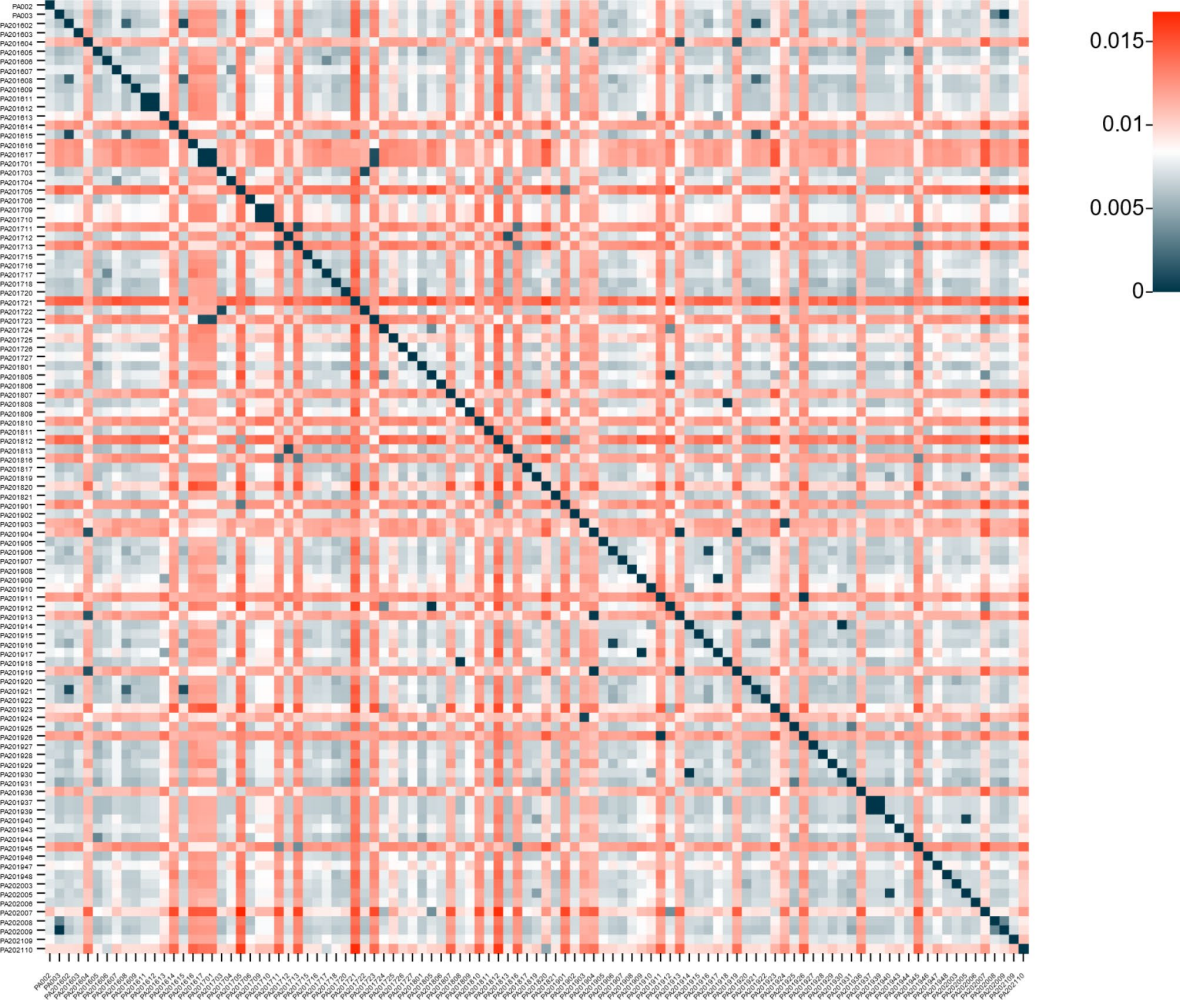
Heatmap representing interspecies a mash distance, the mash distances between these genomes are all less than 0.015, which represents that the ANI of these strains is greater than 95%, same as *P. aeruginosa*. Both horizontal and vertical axes are the names of *P. aeruginosa* strains in this study

A mash distance of 0 means that the genomes of the bacteria are identical, a mash distance of 1 means that the genomes of the k-mers are not shared, and a mash distance less than or equal to 0.5 is approximately equal to 95% average nucleotide identity (ANI) and 70% DNA-DNA hybridization (Fig. 7).

### Classification, number and amino acid sequence of variants of CrpP

A total of 47 *crpP* genes, in 43 *P. aeruginosa* strains, were predicted after abricate prediction analysis. Reference to the classification according to Zhichen Zhu [31]. In total, 17 CrpP variants were identified, including nine new variants of 1.1, named 1.33, 1.34, 1.35, 1.36, 1.37, 1.38, 1.39, 1.40, 1.41, and one CrpP variant with a higher degree of variation, named 7.1.

**Fig. 8:**
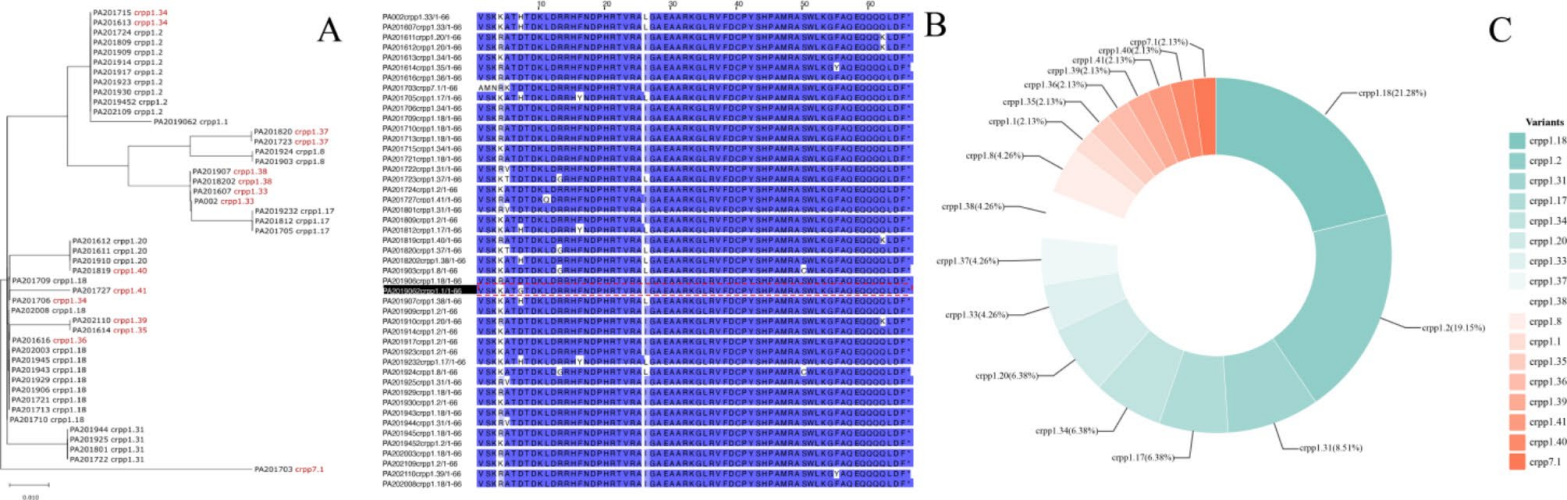
**A**: Those marked in red are newly discovered variants in this study, and evolutionary trees were drawn using the Maximum Likelihood Tree method of MEGA X, with bootstrap set to 1000; **B**: Jalview was used to compare the 47 CrpP amino acids, that marked in the red box is CrpP1.1; **C**: The top five CrpP in terms of number are CrpP1.18 (10/47), CrpP1.2 (9/47), CrpP1.31 (4/47), CrpP1.17 (3/47), CrpP1.34 (3/47). In addition to this, we identified transposons encoding both CrpP in four *P. aeruginosa* strains. The amino acid sequences of the CrpP variants newly identified are listed in Attachment 3

With the exception of CrpP1.1, the amino acid ranked seventh is the one with the highest frequency of variants, with (7/47) mutating to H and (39/47) amino acids mutating to D. The second most frequent mutation is in the fourth position, K, mutated to R, in this 24 CrpP amino acid sequence. Compared to CrpP1.1, CrpP7.1 has a mutation in the first five amino acids, which is where it differs most from other CrpP1.1 amino acids (Fig. 8).

### Comparison of transposon gene clusters carrying novel variants of CrpP

**Fig. 9:**
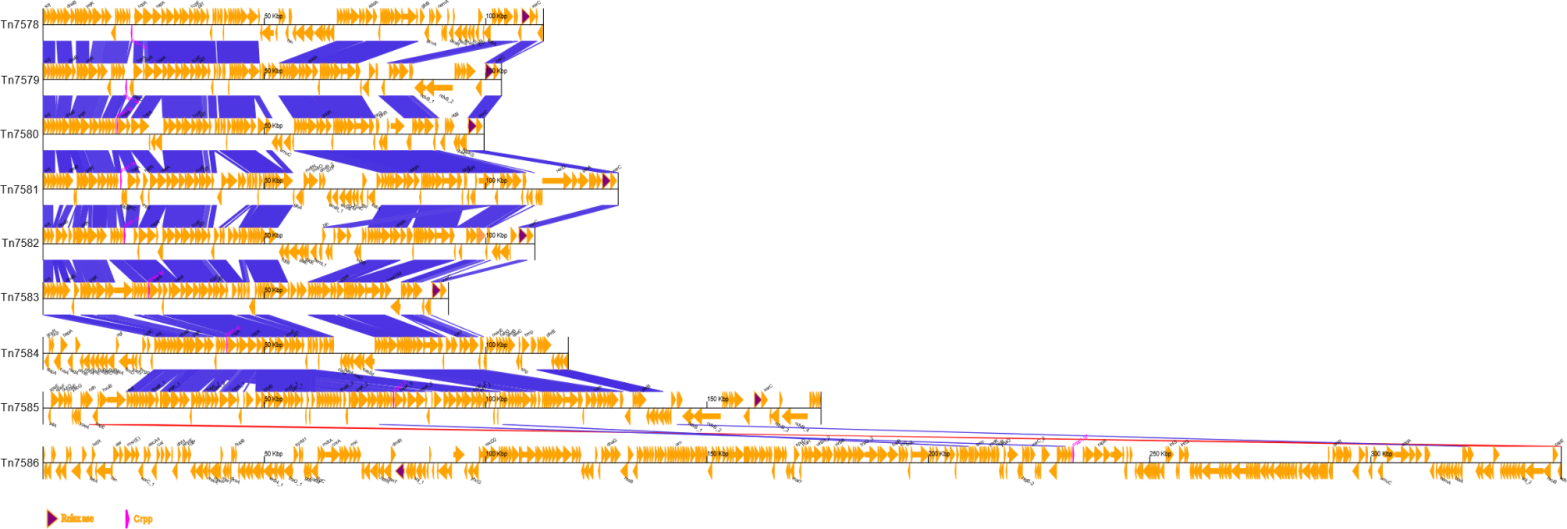
The gbk file was used for comparison and the comparison option was multiple nucleic acid to nucleic acid. the gene annotated by prokka was selected as the label to be marked out; CrpP variants are shown in pink. With the exception of the longest Tn7586, Tn7578-Tn7585 are 101 kb-179 kb in length

PA201607, PA201613, PA201614, PA201616, PA201727, PA202110, PA201819, PA201703, PA201820 carry transposons Tn7578-Tn7586 respectively. Among the 9 variants mentioned above, Tn7586 (PA201820) has a total length of 349 kb and differs greatly from the other transposons. In addition to carrying the *crpP* gene, it also carries the *tet(A)*, *msr(E)*, *mph(E)*, *qnrVC6*, *aac(6’)-IIa*, *catB11*, *dfrA1*, and *sul1* genes. Tn7585 carries variant 7.1 with a nucleotide length of 179 kb, second in length only to Tn7586 (Fig. 9).

### Genome accession numbers and transposon numbers

BioProject ID number submitted on NCBI is PRJNA945332. The sequences carrying the newly identified CrpP variant transposons in this study were registered on the transposon registry (https://transposon.lstmed.ac.uk/), generating the accession numbers Tn7578 - Tn7586 [34].

## Discussion

In this study, 101 human-derived *P. aeruginosa* strains isolated from Jiangsu Province, China, were subjected to bacterial antimicrobial resistance studies and 103 *P. aeruginosa* strains were subjected to whole-genome analysis including ST types, serotypes and resistance genes, etc.

In this study, MICs were determined for a total of 12 antibiotics from the six major classes of antibiotics recommended for use by CLSI, including aminoglycosides, beta-lactams, monocyclic lactams, cephalosporins, fluoroquinolones, and carbapenems. The sensitivity rates for colistin, amikacin, gentamicin, ceftazidime, piperacillin, piperacillin-tazobactam, ciprofloxacin, meropenem, aztreonam, imipenem, cefepime and levofloxacin were 100%, 95.15%, 86.41%, 72.82%, 71.84%, 69.90%, 55.34%, 52.43%, 50.49%, 50.49%, 49.51% and 47.57% respectively. Meanwhile, a total of 31 strains were MDR strains, and the carriage rate of MDR strains was 30.69% (31/101). A research conducted by European Centre for Disease Prevention and Control in 2015 showed that 13.7% of *P. aeruginosa* were resistant to at least three major classes of antibiotics. Our study showed that the *P. aeruginosa* strains isolated from Jiangsu Province had a higher MDR occurrence rate than those from European countries. Besides, *P. aeruginosa* that carry inherent mechanisms of resistance to antibiotic have also been monitored, the results showed that *P. aeruginosa* exhibited high levels of resistance to eight antibiotics include ampicillin, ampicillin-sulbactam, cefazolin, ceftazidime, chloramphenicol, moxifloxacin, tetracycline, and trimethoprim-methoxybenzazole .

The analysis of ST research shows that with the exception of the unidentifiable ST type, ST244 (5/103) was found in all four years respectively, and the next most frequent ST type was ST1076 (4/103). Overall, these 103 clinical *P. aeruginosa* isolates had a wide variety of ST types, with a total of 50 ST types identified. High-risk ST clones are usually associated with virulence, transmission, and MDR/XDR of bacteria. Several investigations have shown that *P. aeruginosa* strain ST244 is associated with *bla*_GES_, *bla*_IMP_, *bla*_KPC_, *bla*_VIM_ [4]. Meanwhile, serotyping of the *P. aeruginosa* strains in the present study showed that serotypes included O6, O11, O2 and O1, accounting for 29.13% (30/103), 23.30% (24/103), 18.45% (19/103) and 11.65% (12/103) respectively, these four serotypes together account for more than 80% of the total. Between 2005 and 2017, the most prevalent serotypes of *P. aeruginosa* found in 413 patients from 10 countries on four continents were O11 (*n* = 89; 22%), O1 (*n* = 58; 14%) and O6 (*n* = 53; 13%) [32]. A survey of 1,445 *P. aeruginosa* strains conducted in Spain in 2017 found that the most common serotypes were O6 (17.8%), O1 (15.4%) and O11 (13.3%) [8]. Overall, our study showed that the predominant serotypes of *P. aeruginosa* isolates from hospitals in Jiangsu Province, China, were not significantly different from the predominant serotypes reported in various other countries.

For the analysis of virulence genes, the cytotoxic exoenzyme was chosen to focus on. Among the cytotoxic exoenzyme, ExoU has been reported to significantly enhance the virulence of *P. aeruginosa* [33]. Among the various phenotypes of *P. aeruginosa* isolates, clinical isolates of serotype O11 were found to secrete ExoU, more frequently than other serotypes and serotype O11 was associated with increased lung injury in a mouse model of pneumonia [34]. It is note worthy that our study shows the O11 serotype accounts for 50% of the ExoU positive strains, which is significantly higher than the percentage of ExoS, ExoY and ExoT positive *P. aeruginosa* strains. Most *P. aeruginosa* strains secrete either ExoS or ExoU, but not both. It is rare for a single strain of bacteria to carry both ExoU and ExoS [35]. In our collection of 103 human-derived *P. aeruginosa* strains, only one strain was present with both ExoU and ExoS.

In the analysis of antimicrobial resistance gene, *aph(3’)-IIb* (103, 103/103), *fosA* (103, 103/103), and *catB7* (102, 102/103), which mediated resistance to aminoglycosides, fosfomycin, and chloramphenicol, respectively, and were found most frequently. Carbapenem antibiotics are commonly used for the treatment of MDR *P. aeruginosa* infections, and carbapenemase-producing resistance genes such as *bla*_GES−1_, *bla*_GES−14_, and *bla*_VIM−2_ are also predicted in the monitoring of resistance genes. Besides, quinolones are considered to be common agents in the treatment of *P. aeruginosa* infections. Among the PMQR genes that mediate resistance to quinolones, *qnrB52* (PA201944), and *qnrVC6* (PA201820), were also predicted to present resistance to ciprofloxacin as resistant and mediated, respectively.

Another gene of interest, *crpP* remains controversial as it is presumed to be transferable and resistant to ciprofloxacin. In the analysis of resistance genes, *crpP* was found to have many nucleotide mutations, and then its amino acid mutant variants continued to be investigated and classified based on the article published by Zhichen Zhu [31]. Nine new derivative variants of CrpP1.1 were identified, named 1.33, 1.34, 1.35, 1.36, 1.37, 1.38, 1.39, 1.40, 1.41 and one variant of CrpP with a higher degree of variability, named 7.1. In addition to this, transposons encoding both CrpP were identified in four strains of *P. aeruginosa*. 47/103 CrpP variants were identified in this study, representing 45.63%. The top five CrpP in terms of number are CrpP1.18 (10/47), CrpP1.2 (9/47), CrpP1.31 (4/47), CrpP1.17 (3/47), CrpP1.34 (3/47). In a separate survey of CrpP variants of *P. aeruginosa*, strains were isolated mainly from patients in eastern China, and a total of 117 CrpP variants were found in 200 *P. aeruginosa* strains, representing a total of 58.5% [31].

With the exception of CrpP1.1, the amino acid in the seventh position is the amino acid that appears most frequently as a variant, with (7/47) mutating to H and (39/47) amino acids mutating to D. The second most frequent mutation is in the fourth position, K, which mutates to R and occurs in 24 CrpP amino acids. Compared to CrpP1.1, CrpP7.1 has a mutation in the first five amino acids, which is where it differs most from CrpP1.1. The CrpP protein has 66 amino acids and is more conserved in the N-terminal region, with only amino acids 1.20 and 1.40 mutated to K at position 62, presumably with residues important to its function in the N-terminal region. Meanwhile, only 10 of the 47 strains carrying the CrpP variant were resistant to ciprofloxacin, carrying variants classified as CrpP1.2, CrpP1.4, CrpP1.8, CrpP1.18, CrpP1.31, CrpP1.36, CrpP1.39. The effect of mutations at different positions of the CrpP amino acid on quinolones is still worth exploring.

In this research, we monitored *P. aeruginosa* for ST type and serotype, as well as antimicrobial resistance genes and virulence genes, which increased our knowledge of strains with highly combined competence (highly pathogenicity, strong antimicrobial resistance, high-risk ST type, and serotypes with highly antimicrobial resistance/virulence), and were used to predict the epidemiological trend of *P. aeruginosa*. At the same time, we conducted antibiotic susceptibility testing of bacteria, which is of guiding value for clinical medication. In summary, we can accurately characterize them in many ways in order to develop global strategies to combat them.

## Conclusions

In this study, 103 strains of *P. aeruginosa* isolated from China between 2016 and 2021, were studied for drug resistance, resistance genes, prevalent serotypes, ST types and pan-genome analysis with a focus on the transferability of CrpP-carrying variants and the structure of their variants, enriching the epidemiological data on clinical *P. aeruginosa*.

### Electronic supplementary material

Below is the link to the electronic supplementary material.

**Supplementary Material 1:** Bacterial information in this article

**Supplementary Material 2:** Statistics of antibiotic susceptibility test results for the strains in this article

**Supplementary Material 3:** CRPP variant statistics and ST-type statistics

## Data Availability

The data sets supporting the results of this article are included within the article and its additional files.
